# Recognition memory across the lifespan: the impact of word frequency and study-test interval on estimates of familiarity and recollection

**DOI:** 10.3389/fpsyg.2013.00787

**Published:** 2013-10-30

**Authors:** Beat Meier, Alodie Rey-Mermet, Nicolas Rothen, Peter Graf

**Affiliations:** ^1^Institute of Psychology and Center for Cognition, Learning and Memory, University of BernBern, Switzerland; ^2^Department of Psychology and Sackler Centre for Consciousness Science, University of SussexBrighton, UK; ^3^Department of Psychology, University of British ColumbiaVancouver, BC, Canada

**Keywords:** aging, development, episodic memory, recognition memory, lifespan

## Abstract

The goal of this study was to investigate recognition memory performance across the lifespan and to determine how estimates of recollection and familiarity contribute to performance. In each of three experiments, participants from five groups from 14 up to 85 years of age (children, young adults, middle-aged adults, young-old adults, and old-old adults) were presented with high- and low-frequency words in a study phase and were tested immediately afterwards and/or after a one day retention interval. The results showed that word frequency and retention interval affected recognition memory performance as well as estimates of recollection and familiarity. Across the lifespan, the trajectory of recognition memory followed an inverse u-shape function that was neither affected by word frequency nor by retention interval. The trajectory of estimates of recollection also followed an inverse u-shape function, and was especially pronounced for low-frequency words. In contrast, estimates of familiarity did not differ across the lifespan. The results indicate that age differences in recognition memory are mainly due to differences in processes related to recollection while the contribution of familiarity-based processes seems to be age-invariant.

## Introduction

Evidence from developmental and cognitive aging studies suggests that episodic memory follows an inverted U-shape function with an increase in performance from childhood to adolescence, a peak in young adulthood, followed by a steady decline in later life. Episodic memory refers to the memory of one's past, the “when,” “where,” and “what” of particular events one has experienced (e.g., Tulving, 2002). It is an important ability, which permits us to keep track of our life history and to distinguish new from old information. It is likely that different mechanisms are responsible for the performance rise and fall across the lifespan, but the development of overarching theoretical models has just begun (e.g., Graf and Ohta, 2002; Fingerman et al., 2011; for overviews). Nevertheless, there is general agreement that episodic memory has an active subjectively-controlled component which is engaged, for example, for recollecting what was said on a particular occasion, as well as a more passive autonomous component which seems involved in deciding whether a particular stimulus has been encountered before that is, whether it is “old” or “new.” Both of these components seem to influence performance on recognition memory tests which is the focus of this article.

In a typical recognition memory experiment, words are presented in a study phase. Later, in a test phase, these words are presented again, intermixed with new words that have not been exposed previously, and participants are required to indicate for each word whether it is “old” or “new.” A correct decision can occur as a consequence of recollecting the memory of that word as it was presented in the study phase (i.e., recollection) or due to more fluent processing which is attributed to the oldness of an item (i.e., familiarity). Recollection is assumed to engage processes which are more resource demanding and slower than familiarity (e.g., Hintzman and Caulton, 1997; Joordens and Hockley, 2000; Yonelinas, 2002).

In this article, we first present a brief overview of contemporary models of lifespan recognition memory development. We use these models to introduce the rationale and the hypotheses for the present study which investigated the trajectory of recognition memory performance across the lifespan. To our knowledge, this is the first study which used the same recognition task to examine performance across such a large segment of the lifespan. In three separate experiments, we investigated the impact of word-frequency and of different retention intervals in five cohorts ranging from 14 up to 85 years of age, allowing us to analyze the trajectory of performance on recognition memory hit and false alarm responses, as well as to obtain estimates of recollection and familiarity.

### Lifespan approaches

Only a few theoretical accounts are available for explaining lifelong changes in cognitive abilities (e.g., Baltes, 1987; Salthouse, 1996; Craik and Bialystok, 2006; Li and Baltes, 2006; Shing and Lindenberger, 2011). Perhaps the most prominent of these is Salthouse's (1985, 1996) account which focuses on the speed of cognitive operations. According to his account, the speed with which many cognitive processes can be executed increases from infancy to young adulthood and then declines from the twenties to old age, and it is assumed that the general or global slowing which occurs in late adulthood is the primary cause of age-related declines in cognition. Salthouse suggested that slowed processing could lead to cognitive deficits by two distinct routes, which he called a *limited time mechanism* and a *simultaneity mechanism*. The limited time mechanism captures the idea that insufficient time might be available for some operations when more or most of the available time is required for completing initial or precursor operations. In the context of a conversation, for example, there may not be sufficient time for reflecting on the full implications of a message because most of the available time is spent on the initial sensory and perceptual encoding of the message. Salthouse used the simultaneity mechanism to discuss the fact that slowing might reduce the pool of information that is available simultaneously (i.e., at the same time), because the products of earlier processing operations may have been lost before later processing operations are completed.

Craik and Bialystok (2006) proposed a view of lifespan differences in cognitive processing which focuses on the development of knowledge representations and of cognitive control. By this view, representations are defined as crystallized knowledge structures or schemas, which serve as the scaffolding for encoding and retrieving episodic memories and as the knowledge base for making predictions about common events. By contrast, cognitive control refers to the processes which we operate on the knowledge structures, for example, by choosing the best schema for making predictions about a particular event or by using a schema to guide recollection of critical components of a recently experienced event. According to Craik and Bialystok, representations and control and their manner of interacting with each other evolve across the lifespan. Representational knowledge is assumed to increase rapidly during childhood, to continue accumulating throughout adulthood and to remain relatively stable in old age. In contrast, cognitive control is also assumed to increase from childhood to young adulthood, but to follow a steady decline thereafter. Craik and Bialystok noted that these developmental patterns are largely compatible with the developmental maturation of different brain areas, in particular the medial temporal lobe (MTL) and the prefrontal cortex (PFC). It is assumed that the development of MTL areas underlies the build-up and functionality of knowledge representations, while the developmentally later maturation and earlier attrition of prefrontal areas mediates the functionality of cognitive control.

Building on the framework of lifespan development introduced by Baltes and colleagues (Baltes, 1987; Baltes et al., 2006), Shing and Lindenberger (2011) have made a distinction between associative and strategic components in order to account for episodic memory performance across the lifespan. Associative components are assumed to be involved in binding different aspects of an event during encoding while strategic components are used to elaborate relational aspects on the basis of existing semantic knowledge during encoding and retrieval (see Shing et al., 2008). It is assumed that the associative component mostly relies on structures of the medial temporal lobes (MTL) while the strategic component depends primarily on the prefrontal cortex (PFC). Given that, the development of the MTL and the PFC follow different trajectories, differential hypotheses can be derived regarding the contribution of the associative and strategic components to memory performance at different phases of the lifespan (cf. Craik and Bialystok, 2006). For example, in agreement with the stronger decline of PFC functions compared to MTL function in old age, older adults engage less in strategic encoding and retrieval operations and this is assumed to account for the performance decline in episodic memory performance.

### Recollection and familiarity across the lifespan

None of the foregoing accounts of lifespan changes in cognition has focused on the distinction between recollection and familiarity which is a fundamental building block of many recent accounts of age-related declines in episodic memory performance. Recollection, assumed to depend on controlled processing and strategic elaboration, refers to the mental reinstatement of previously experienced events that gives rise to memories that are vivid and rich in contextual details. By contrast, familiarity refers to the subjective impression that an event has been experienced before, and more particularly, to a feeling of “I know this” or “I have experienced this before” which typically arises in the absence of any recollection of contextual information about the same event. Although the distinction between recollection and familiarity was not included in the lifespan models described in the preceding section, this distinction seems compatible especially with the view proposed by Craik and Bialystok (2006) as well as with the proposal of Shing and Lindenberger (2011). In addition, the distinction between recollection and familiarity also seems to map closely onto the distinction between controlled and automatic processing which has been offered as a framework for understanding adult age-related changes in episodic memory (Light et al., 2000; Prull et al., 2006). In an in-depth review article, Yonelinas (2002) summarized several other models of recognition memory which postulate recollection as an all-or-none process and familiarity as a continuous process, while stipulating that these two make independent contributions to recognition memory performance.

Although a variety of different methods have been used for estimating the relative contributions of recollection and familiarity to recognition memory performance, they tend to lead to similar results and conclusions. For the present study, we relied primarily on the remember/know procedure which was originally introduced by Tulving (1985). This procedure requires participants in a recognition memory test to augment their “old” decisions with an additional judgment, indicating whether the test item was recognized as old based on the recollection of contextual details from the study phase or based on the familiarity of the item in the absence of recollection. The first of these judgments, called “remember” responses, are used to estimate the influence of recollection on performance while the second type, called “know” responses, are treated as revealing the contribution due to familiarity.

Developmental studies have revealed that recognition memory performance increases with age, and it appears that this increase is due primarily to recollection (i.e., remember responses), in the absence of age differences due to familiarity. This pattern of results was reported by Billingsley et al. (2002) in a study which included participants between 8 and 19 years of age. Similarly, in a sample of 6 and 24 year old participants, Ofen et al. (2007) found a significant correlation between age and remember responses, but not between age and know responses. Likewise, in a sample of 6–18 year olds, Ghetti and Angelini (2008) found a similar pattern of results, using confidence judgments combined with the analysis of receiver operating characteristics to estimate recollection and familiarity (ROC; cf. Yonelinas, 2002).

However, in a study that investigated the lifespan trajectory of the event-related potentials underlying recollection and familiarity, Friedman et al. (2010) found that 9–10 year old children recruited less familiarity-based processes compared to 13–14 year old children, young, and older adults. In contrast, older adults recruited less recollection based processes compared to the younger groups. Moreover, Mecklinger et al. (2010) found that the ERP correlate of recollection can be reliably recorded in 8 year old children, and they suggested that their recollection is already fully developed, but that their recognition memory network is still weaker and less matured.

Changes in recollection in combination with stability in familiarity are the typical pattern that has emerged from studies with older adults. Episodic memory performance is correlated negatively with age, and age-related declines are typically larger on recall than recognition memory tests (Craik and McDowd, 1987; Meier et al., 2002). In recognition memory, the age-related declines that have been observed are mostly due to lower levels of recollection, although some studies have also revealed an age-related decline in familiarity estimates. In all cases, the latter is less pronounced than the age-related decline in recollection (Parkin and Walter, 1992; Mäntylä, 1993; Perfect et al., 1995; Java, 1996; Norman and Schacter, 1997; Perfect and Dasgupta, 1997; Schacter et al., 1997; Mark and Rugg, 1998; Friedman and Trott, 2000; Clarys et al., 2002; Lövdén et al., 2002; Bastin and Van der Linden, 2003; Bunce, 2003; Comblain et al., 2004; Bunce and Macready, 2005; Duarte et al., 2006, 2008; Prull et al., 2006; Bugaiska et al., 2007; Skinner and Fernandes, 2009; Friedman et al., 2010). Different interpretations have been invoked to explain these somewhat discrepant results. For example, it has been argued that the differences are due to the particular measurement method that was used (i.e., the process dissociation procedure seems to be less prone to show deficits in familiarity estimates than the remember/know procedure; Light et al., 2000; Prull et al., 2006). Differences might also depend on the overall levels of performance, with studies reporting high estimates of recollection producing age effects on estimates of familiarity. However, it has been argued that this effect is spurious and rather related to potential ceiling effects (Yonelinas, 2002).

### The present study

The present study was designed to investigate the lifespan trajectory of recognition memory test performance, and especially the distinct influences due to recollection and familiarity. The study included two critical variables—word frequency and study-test interval—that are known to have different effects on recollection and familiarity. Specifically, the goal of this study was to test the hypothesis that these variables may have a differential effect on the lifespan trajectory of recognition memory performance and its underlying processes.

Previous research shows that on recall tests, high-frequency words are remembered better than low-frequency words, suggesting an advantage for recollection (Gregg et al., 1980). In contrast, on recognition tests, the reverse pattern emerges (Glanzer and Adams, 1985). Because high-frequency words have higher baseline familiarity compared to low-frequency words, new high-frequency words are more likely to produce familiarity-based false alarms than new low-frequency words. Low-frequency words are assumed to have fewer contextual associations than high-frequency words and thus their situation-specific activation during the study phase of an experiment is assumed to result in the formation of more distinctive memory traces. Therefore, compared to high-frequency words, the recognition of low-frequency words is typically advantaged and results in a higher hit rate. For this reason, the hit rate for low-frequency words is typically used to estimate recollection, while the false alarm rate for high-frequency words is assumed to provide an estimate of familiarity (Reder et al., 2000). Because hit rates (i.e., correct recognition of old, previously presented items) are increased and false alarm rates (i.e., incorrect “recognition” of new, not previously presented items) are decreased when performance of low-frequency words is compared to performance of high-frequency words, this effect is also referred to as the word-frequency mirror effect (Glanzer and Adams, 1990).

Both recollection and familiarity are typically greater for low compared to high-frequency words, but the effect due to frequency tends to be larger on recollection than on familiarity (Gardiner and Java, 1990; Kinoshita, 1995; Gardiner et al., 1997; Guttentag and Carroll, 1997; Joordens and Hockley, 2000; Reder et al., 2000; Hirshman et al., 2002). This pattern of results is typically found with young adult participants. However, in a cognitive aging study, Balota et al. (2002) found an age-related decline in the hit rate for low-frequency words, but not for high-frequency words while there was a slight age-related increase in false alarms for both low- and high-frequency words. This result is consistent with the proposal that there is an age-related decline in recollection while familiarity remains stable. However, as Balota et al. did not include remember/know judgments to test whether word-frequency and age have an interactive effect their specific contribution to familiarity and recollection remain to be determined. One goal of the present study was to fill this gap.

A further goal of the present study was to test the impact of a study-test delay (i.e., the retention interval) on recognition memory performance as well as on estimates of familiarity and recollection. As reviewed by Yonelinas (2002), different theoretical predictions exist. According to models that assume that familiarity reflects a temporary activation of an item in the knowledge representation system, it would be expected to decrease rapidly (e.g., Mandler, 1979). In fact, over the very short-term (i.e., seconds to a few minutes) the empirical results agree with this notion (e.g., Hockley, 1991, 1992). However, across longer time intervals (i.e., weeks to several months) familiarity seems to remain rather stable while there is a substantial decrease in recollection (e.g., Gardiner and Java, 1991; Hockley and Consoli, 1999). Wilson et al. (1983) investigated the word frequency effect either immediately or after an interval of 1 week to examine whether, after the delay, healthy controls would show a similar lack of frequency effect as evidenced on immediate tests by individuals with dementia. However, this was not the case. There was still a word-frequency effect after the 1 week interval. As they did not include remember/know judgments, however, it is not clear whether the relative contribution of recollection and familiarity changed across test sessions. Joordens and Hockley (2000) compared a test condition in which each of ten 24-item study list was immediately tested and in which remember/know responses were collected with a delayed condition that involved testing at the end of the session. Their results showed main effects of word frequency and study-test delay, but no interaction for hits and false alarms. They concluded that the impact of word-frequency and test delay on remember/know judgments is additive.

Consistent with these results, we hypothesized that test delay would affect both recollection and familiarity, that test delay and word frequency would both interact with age particularly for recollection, and that no triple interaction between age, word frequency and retention interval would emerge, neither for familiarity nor for recollection. In the present study we manipulated word-frequency and study-test interval across five age groups in order to test their influence on recognition memory performance and on the estimates of recollection and familiarity. In general, we expected to find an inverse u-shape lifespan trajectory. Moreover, we hypothesized to find a more pronounced u-shape function for low-frequency words than for high-frequency words for both recognition memory performance and estimates of recollection, but not for estimates of familiarity. While we expected a performance decline across the retention interval, we were particularly interested to test whether this pattern would flatten out more for high than for low-frequency words and whether this potential change in performance would be different across the lifespan.

#### General method

All three experiments used the same general method which is described here, while features unique to each experiment are reported in connection with each experiment.

***Participants***. The experiments included participants from five age groups: Children (aged 14–16), young adults (aged 25–30), middle-aged adults (aged 45–50), young-old adults (aged 65–70), and old-old adults (aged above 75). The experiments were conducted in the context of a research method class at the Swiss Distance University. Each student had to recruit and test participants from each age group. Inclusion criteria were German as first language, normal or corrected to normal vision, and self-rated good health. In order to prevent the accidental inclusion of individuals afflicted with age-related pathologies, participants from both older groups (young-old and old-old) had to achieve a score of at least 27 on Mini Mental State Examination (Folstein et al., 1975).

***Materials***. The study and test lists were composed according to the method of Balota et al. (2002; cf. Weiermann et al., 2010). High- and low-frequency words were selected from the word database of the University of Leipzig (http://wortschatz.uni-leipzig.de). The words ranged from 3 to 9 letters and were matched in letter-length across frequency categories. For the study and test phase of each experiment, words were displayed in the center of a computer screen, in white 60-point Times New Roman font against a black background.

***Design***. The basic design of each experiment had three factors. For all experiments, age group was a between-subjects factor while word-frequency was manipulated within-subject. In Experiment 1, retention interval was manipulated between-subjects, while in Experiments 2 and 3, it was manipulated within-subject.

***Procedure***. Each experiment consisted of a study phase and a test phase and participants performed both phases individually, under the direct supervision of the experimenter. In the study phase, participants were informed that they would see words presented on a computers screen one at a time. They were instructed to read each word aloud and to remember it for a later memory test. To verify comprehension of the instructions, participants were asked to summarize them for the experimenter. For the study phase, each word was displayed for 2 s. Then, the experimenter pressed a key to advance to the next word. Immediately after the study phase, there was a 20-min filled delay during which participants completed a set of unrelated questionnaires, including the Mehrfachwahl-Wortschatztest, a German equivalent to the National Adult Reading Test (Lehrl et al., 1991) to assess verbal intelligence.

For the test phase, participants were informed that they would see more words, some of them from the study phase (i.e., old words) and some which had not been displayed before (i.e., new words). They were instructed to indicate for each word whether it was old or new. After a “new” decision, the next word appeared immediately. However, after entering an “old” decision, participants were asked to make a Remember or Know judgment in Experiments 1 and 2, or a Remember, Know, or Guess judgment in Experiment 3, following the Method of Weiermann et al. (2010). Specifically, the participants were instructed to make a Remember response when they were able to recollect the word from the study episode, to give a Know response when they were not able to recollect the word, but nevertheless believed that they had studied it before, and to give a Guess response when they only guessed that they had studied it before. To collect the Remember/Know and Remember/Know/Guess responses, the experimenter pressed the appropriate key on the keyboard, and upon doing so, the next word was displayed. At the end of the test session, the Mini Mental State Examination (Folstein et al., 1975) was completed by each participant from the young-old and old-old groups.

***Data analysis***. For each participant, hit rates, false alarm rates and the discrimination score *Pr* (i.e., hits minus false alarms; Snodgrass and Corwin, 1988) were computed for each frequency category and condition. Based on remember/know judgments, estimates of recollection and familiarity were computed with the formulae by Yonelinas et al. (1998): Recollection = [(Remember old − Remember new)/(1 − Remember new)]; Familiarity = [z(Familiarity old) − z(Familiarity new)], with Familiarity old = [Know old/(1 − Remember old)] and Familiarity new = [Know new/(1 − Remember new)].

An alpha level of 0.05 was used for all statistical tests. Effect sizes are expressed as partial η^2^ values.

## Experiment 1

### Method

#### Participants

A total of 186 individuals (37 children, 36 young adults, 36 middle-aged adults, 37 young-old adults, and 40 old-old adults) participated in the study. Two of the young-old adults and three of the old-old adults had to be excluded because they obtained a MMS value below 27 points. Demographic characteristics of the final sample are presented in Table 1.

**Table 1:** **Descriptive characteristics of the samples for each experiment**.

	***n***	**Age (years)**	**Male-female ratio**	**Years of education**	**Estimate of verbal IQ**
**EXPERIMENT 1**					
Children	37	14.8	57:43	8.7	101.6
Young adults	36	27.7	47:53	15.3	122.1
Middle-aged adults	36	46.9	39:61	16.5	126.9
Young-old adults	35	66.8	46:54	14.1	130.9
Old-old adults	37	79.1	30:70	12.4	117.7
**EXPERIMENT 2**					
Children	30	15	40:60	8.8	101.2
Young adults	30	26.9	30:70	16	118.3
Middle-aged adults	30	47.2	47:53	16	121.4
Young-old adults	28	67	43:57	12.9	121.4
Old-old adults	24	79.9	58:42	13.9	120.5
**EXPERIMENT 3**					
Children	39	14.5	49:51	8.2	95.2
Young adults	38	28.1	29:71	15.5	111.2
Middle-aged adults	40	46.3	35:65	15.2	120.8
Young-old adults	35	66.6	34:66	13.7	117.4
Old-old adults	34	79.6	32:68	12.5	108.5

#### Materials

Materials were 48 high-frequency and 48 low-frequency words. According to the vocabulary database of the University of Leipzig (http://wortschatz.uni-leipzig.de), their mean frequency class was 15 (*SD* = 1.5) for low-frequency words and 8.9 (*SD* = 1.5) for high-frequency words, *t*_(94)_ = 20.01, *p* < 0.001. A random half of the items from each frequency condition was chosen to form the study list, with the remainder used as new items on the recognition test. The assignment of words to the study list was counterbalanced across participants such that each word occurred both as an old and a new item on the recognition test. The assignment of words to the recognition test was also counterbalanced across participants such that each word occurred in the immediate as well as in the delayed tests.

#### Procedure

The procedure was as described in the General Method section. For half of the participants, the test phase started immediately after the filled delay (immediate test); for the other half, it started 24 h later (delayed test).

### Results

**Figure 1:**
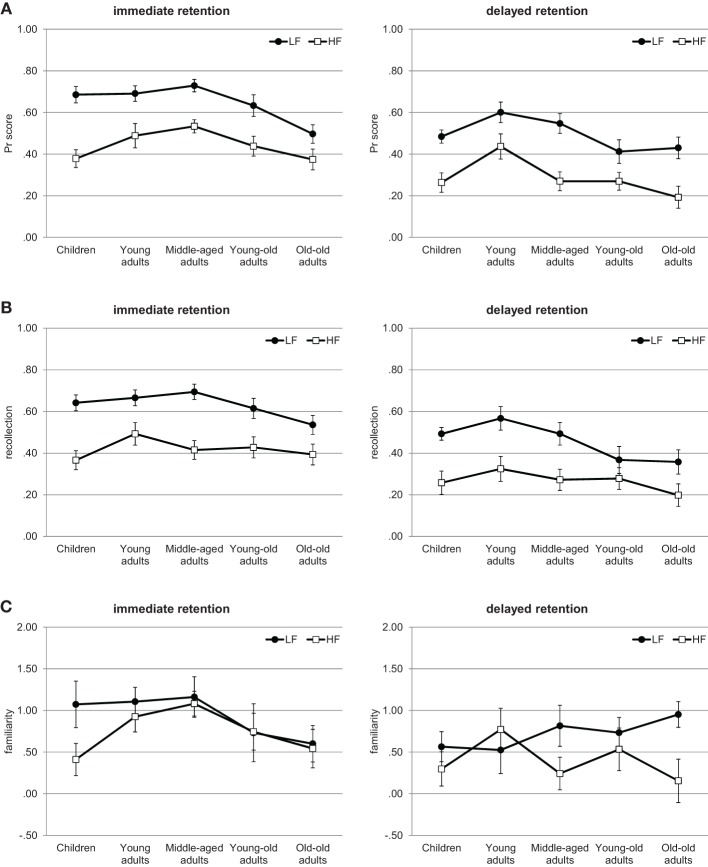
**Experiment 1**. Recognition memory performance across the lifespan. **(A)** *Pr* scores. **(B)** Estimates of recollection. **(C)** Estimates of familiarity. Error bars represent standard errors. LF, low-frequency words; HF, high-frequency words.

The main focus of the analyses is on recognition memory performance *Pr* and on the estimates of recollection and familiarity. These results are depicted in Figure 1, and in order to facilitate comparison with other investigations, hits and false alarm rates are also presented in Table 2. The results show a consistent and prominent word-frequency effect, which was borned out by the statistical analyses presented in the Supplementary Materials.

**Table 2:** **Experiment 1: Means and standard errors of hits and false alarm rates**.

		**Children**				**Young adults**				**Middle-aged adults**				**Young-old adults**				**Old-old adults**			
		**Immediate**		**Delayed**		**Immediate**		**Delayed**		**Immediate**		**Delayed**		**Immediate**		**Delayed**		**Immediate**		**Delayed**	
		***M***	***SE***	***M***	***SE***	***M***	***SE***	***M***	***SE***	***M***	***SE***	***M***	***SE***	***M***	***SE***	***M***	***SE***	***M***	***SE***	***M***	***SE***
**REMEMBER**																					
HIT																					
	LF	0.65	0.04	0.53	0.03	0.68	0.04	0.59	0.05	0.70	0.04	0.54	0.05	0.64	0.05	0.49	0.05	0.58	0.05	0.40	0.06
	HF	0.40	0.04	0.36	0.05	0.53	0.05	0.36	0.06	0.44	0.04	0.34	0.05	0.48	0.05	0.39	0.06	0.48	0.05	0.24	0.06
FA																					
	LF	0.03	0.01	0.08	0.02	0.04	0.02	0.05	0.01	0.04	0.01	0.08	0.02	0.05	0.02	0.17	0.04	0.11	0.03	0.08	0.03
	HF	0.05	0.01	0.13	0.03	0.07	0.02	0.05	0.02	0.04	0.01	0.09	0.02	0.10	0.03	0.17	0.05	0.15	0.04	0.07	0.03
**KNOW**																					
HIT																					
	LF	0.10	0.02	0.14	0.03	0.12	0.02	0.16	0.04	0.11	0.02	0.18	0.03	0.09	0.02	0.21	0.03	0.16	0.03	0.26	0.04
	HF	0.16	0.03	0.22	0.04	0.20	0.03	0.28	0.04	0.27	0.05	0.19	0.03	0.16	0.03	0.23	0.03	0.18	0.03	0.24	0.03
FA																					
	LF	0.03	0.01	0.11	0.02	0.07	0.02	0.10	0.02	0.04	0.01	0.09	0.01	0.04	0.01	0.12	0.02	0.13	0.04	0.15	0.03
	HF	0.12	0.03	0.18	0.02	0.18	0.03	0.14	0.03	0.13	0.03	0.17	0.02	0.10	0.03	0.18	0.03	0.14	0.03	0.21	0.04
**TOTAL**																					
HIT																					
	LF	0.75	0.04	0.67	0.03	0.80	0.03	0.75	0.04	0.81	0.03	0.71	0.04	0.73	0.05	0.70	0.03	0.74	0.03	0.66	0.04
	HF	0.55	0.04	0.58	0.05	0.73	0.05	0.63	0.05	0.71	0.03	0.53	0.04	0.64	0.04	0.62	0.05	0.66	0.05	0.48	0.07
FA																					
	LF	0.07	0.02	0.19	0.03	0.11	0.03	0.15	0.03	0.08	0.02	0.17	0.03	0.09	0.02	0.29	0.04	0.24	0.06	0.23	0.05
	HF	0.17	0.03	0.31	0.04	0.25	0.04	0.20	0.04	0.17	0.03	0.26	0.03	0.20	0.03	0.36	0.06	0.28	0.07	0.28	0.06

M, mean; SE, standard errors; FA, false alarms; LF, low-frequency words; and HF, high-frequency words.

#### Recognition memory performance

For the *Pr* scores, depicted in Figure 1A, a Three-Way analysis of variance (ANOVA) with age group (children, young adults, middle-aged adults, young-old adults, old-old adults) and retention interval (immediate test, delayed test) as between-subjects factors and word-frequency (high, low) as a within-subject factor showed significant main effects of age, *F*_(4, 171)_ = 6.09, *p* < 0.001, η^2^ = 0.12, retention interval, *F*_(1, 171)_ = 35.76, *p* < 0.001, η^2^ = 0.17, and word frequency, *F*_(1, 171)_ = 208.7, *p* < 0.001, η^2^ = 0.55. No interaction reached significance, all *F*s < 1.93, *p*s > 0.11. Overall, recognition memory performance was higher for the immediate than delayed test (*M* = 0.54 and *M* = 0.39, respectively) and for low- than for high-frequency words (*M* = 0.57 and *M* = 0.37, respectively). *Post-hoc* Tukey HSD tests revealed that the age effect was due the lower performance of the old-old adults compared to both the young adults and the middle-aged adults (both *p*s < 0.01); moreover, young-old adults performed lower than young adults (*p* < 0.05). No other group difference reached significance (all *p*s > 0.11).

#### Estimates of recollection

The estimates of recollection, depicted in Figure 1B, were also examined by a Three-Way ANOVA, which showed significant main effects of age, *F*_(4, 171)_ = 2.84, *p* < 0.05, η^2^ = 0.06, retention interval, *F*_(1, 171)_ = 34.45, *p* < 0.001, η^2^ = 0.17, and word frequency, *F*_(1, 171)_ = 181.89, *p* < 0.001, η^2^ = 0.51, as well as a significant interaction between age and word frequency, *F*_(4, 171)_ = 2.67, *p* < 0.05, η^2^ = 0.06. No other interaction reached significance, all *F*s < 1, *p*s > 0.43. Overall, recollection was higher for the immediate than delayed test (*M* = 0.52 and *M* = 0.36, respectively) and for low- than for high-frequency words (*M* = 0.54 and *M* = 0.35, respectively). Separate ANOVAs were used to follow-up the interaction between age and word frequency, and they showed no significant age effect for high-frequency words, *F*_(4, 171)_ = 1.4, *p* = 0.23, η^2^ = 0.03, but a significant age effect for low-frequency words, *F*_(4, 171)_ = 4.46, *p* < 0.01, η^2^ = 0.09. *Post-hoc* Tukey HSD tests for low-frequency words revealed that old-old adults showed lower estimates of recollection than young and middle-aged adults (both *p*s < 0.05); moreover, young-old adults also showed slightly lower estimates of recollection than young adults (*p* = 0.06). No other group difference reached significance (all *p*s > 0.14).

#### Estimates of familiarity

For the estimates of familiarity, depicted in Figure 1C, the Three-Way ANOVA showed significant main effects of retention interval, *F*_(1, 171)_ = 6.16, *p* < 0.05, η^2^ = 0.03, and word frequency, *F*_(1, 171)_ = 7.9, *p* < 0.01, η^2^ = 0.04. There was no age effect and no interaction effects, all *F*s < 1.67, *p*s > 0.16. Overall, familiarity was higher for the immediate than delayed test (*M* = 0.83 and *M* = 0.56, respectively) and for low- than for high-frequency words (*M* = 0.82 and *M* = 0.58, respectively).

### Discussion

In Experiment 1, recognition memory performance showed the expected inverted U-shaped function due to age, the expected decline from the immediate to the delayed test, as well as the performance advantage for low- vs. high-frequency words. The absence of any interaction effects among these variables suggests that they exert an additive influence on performance. Collectively, these findings are consistent with previous research on both age-group differences in recognition memory, as well as with research concerned with word frequency and retention interval effects.

The estimates of recollection showed a different pattern; in addition to the expected effects due to age, retention interval and word frequency, they also revealed a significant interaction between age and word frequency which occurred because the age variable affected recollection of low- but not high-frequency words. As recollection is assumed to depend on elaborative processing, and low-frequency words are more likely to involve this type of processing, one interpretation is that aging is accompanied by a reduction in elaborative processing. In contrast to the estimates of recollection, the estimates of familiarity showed no effect of age and no interaction effects with age as expected. However, they did reveal effects due to word frequency and for the retention interval manipulation.

Overall, Experiment 1 revealed several provoking results. First, for high-frequency words both estimates of recollection and estimates of familiarity did not vary with age. Second, despite the fact that the word-frequency and retention interval manipulations consistently affected recognition memory performance, estimates of recollection and estimates of familiarity, there was a surprising lack of interaction effects, in particular regarding the retention interval manipulation.

In order to increase the statistical power of the latter manipulation, we varied the retention interval within-subject in Experiment 2. By this variation, we were also able to investigate the stability of the estimates of recollection and familiarity across the retention interval. It is noteworthy that differences in the stability of individual differences (i.e., higher stability for recollection compared to familiarity) may be a trivial cause for the differential influence of age on recollection and familiarity. Given that the relationship between a certain measure and any other variable is limited by its reliability, it is clear that the extent to which relationships with other variables can be established is restricted by the stability of that measure itself. It has been shown previously that implicit memory measures—which rely on automatic processes—tend to be less reliable than explicit memory measures—which rely on controlled processes (cf. Meier and Perrig, 2000; Meier et al., 2009). Thus, it is possible that a similar pattern may be present for estimates of recollection and familiarity. In Experiment 2 this question was addressed directly.

## Experiment 2

### Method

#### Participants

A total of 149 participants, 30 children, 30 young adults, 30 middle-aged adults, 29 young-old adults, and 30 old-old adults participated in the study. One of the young-old adults and six of the old-old adults had to be excluded because they failed to achieve the cut-off score of 27 on the MMS. Demographic characteristics of the final sample are presented in Table 1.

#### Materials

Materials were 96 high-frequency and 96 low-frequency words. According to the vocabulary database of the University of Leipzig (http://wortschatz.uni-leipzig.de), the mean frequency class was 17.1 (*SD* = 2.8) for low-frequency words and 8.3 (*SD* = 1.3) for high-frequency words, *t*_(190)_ = 27.93, *p* < 0.001. For each frequency, four different lists were created and administered in a counterbalanced manner across the conditions of the experiment.

Half of the items within each frequency condition were used as “old” items (i.e., two lists of each frequency condition) which were presented in the study phase and the others (i.e., two lists of each frequency condition) were used as “new” items. Half of the study words were used as old words for the immediate test and the other half was used for the delayed test. Similarly, half of the new words were used for the immediate test and the other half were used for the delayed test. The assignment of words to the study and test lists was counterbalanced across participants such that each word occurred equally often in each counterbalancing condition.

#### Procedure

The procedure was as described in the General Method. After the 20-min filled interval, the immediate test phase was administered (immediate test). The participants returned the next day (i.e., after a 24-h retention interval) and then the delayed test was administered.

### Results

Hits and False Alarm rates are presented in Table 3 and the respective analyses are presented in the Supplementary Materials. They showed a consistent word-frequency effect that was slightly smaller in older adults. As in Experiment 1, we focus on recognition memory performance, and on the estimates of recollection and familiarity.

**Table 3:** **Experiment 2: Means and standard errors of hits and false alarm rates**.

		**Children**				**Young adults**				**Middle-aged adults**				**Young-old adults**				**Old-old adults**			
		**Immediate**		**Delayed**		**Immediate**		**Delayed**		**Immediate**		**Delayed**		**Immediate**		**Delayed**		**Immediate**		**Delayed**	
		***M***	***SE***	***M***	***SE***	***M***	***SE***	***M***	***SE***	***M***	***SE***	***M***	***SE***	***M***	***SE***	***M***	***SE***	***M***	***SE***	***M***	***SE***
**REMEMBER**																					
HIT																					
	LF	0.64	0.03	0.42	0.03	0.65	0.03	0.46	0.04	0.72	0.03	0.52	0.04	0.60	0.03	0.41	0.04	0.59	0.04	0.40	0.04
	HF	0.44	0.04	0.27	0.04	0.39	0.04	0.22	0.03	0.49	0.05	0.34	0.04	0.42	0.05	0.29	0.05	0.43	0.05	0.34	0.05
FA																					
	LF	0.04	0.01	0.07	0.02	0.03	0.01	0.05	0.01	0.05	0.02	0.12	0.02	0.09	0.02	0.12	0.02	0.09	0.02	0.17	0.03
	HF	0.05	0.01	0.08	0.02	0.05	0.01	0.05	0.01	0.13	0.03	0.16	0.03	0.13	0.04	0.19	0.04	0.18	0.04	0.19	0.05
**KNOW**																					
HIT																					
	LF	0.12	0.02	0.17	0.02	0.14	0.02	0.19	0.02	0.10	0.02	0.17	0.03	0.13	0.02	0.20	0.02	0.12	0.02	0.18	0.03
	HF	0.16	0.02	0.20	0.02	0.23	0.02	0.28	0.02	0.18	0.03	0.23	0.03	0.20	0.02	0.24	0.03	0.21	0.04	0.25	0.04
FA																					
	LF	0.06	0.01	0.09	0.02	0.05	0.01	0.10	0.02	0.05	0.01	0.11	0.02	0.06	0.01	0.11	0.02	0.07	0.01	0.12	0.02
	HF	0.16	0.02	0.18	0.02	0.15	0.02	0.20	0.03	0.13	0.03	0.23	0.03	0.13	0.02	0.22	0.03	0.17	0.03	0.24	0.04
**TOTAL**																					
HIT																					
	LF	0.76	0.03	0.58	0.03	0.79	0.02	0.65	0.04	0.82	0.03	0.69	0.03	0.73	0.03	0.60	0.03	0.71	0.04	0.58	0.04
	HF	0.59	0.04	0.47	0.04	0.61	0.04	0.50	0.04	0.67	0.04	0.57	0.04	0.62	0.04	0.53	0.04	0.64	0.04	0.58	0.05
FA																					
	LF	0.10	0.02	0.17	0.03	0.08	0.01	0.15	0.02	0.11	0.02	0.23	0.03	0.16	0.02	0.22	0.03	0.16	0.03	0.29	0.04
	HF	0.21	0.02	0.26	0.04	0.20	0.03	0.25	0.03	0.26	0.04	0.39	0.04	0.26	0.04	0.41	0.04	0.35	0.05	0.43	0.06

M, mean; SE, standard errors; FA, false alarms; LF, low-frequency words; and HF, high-frequency words.

#### Recognition memory performance

**Figure 2:**
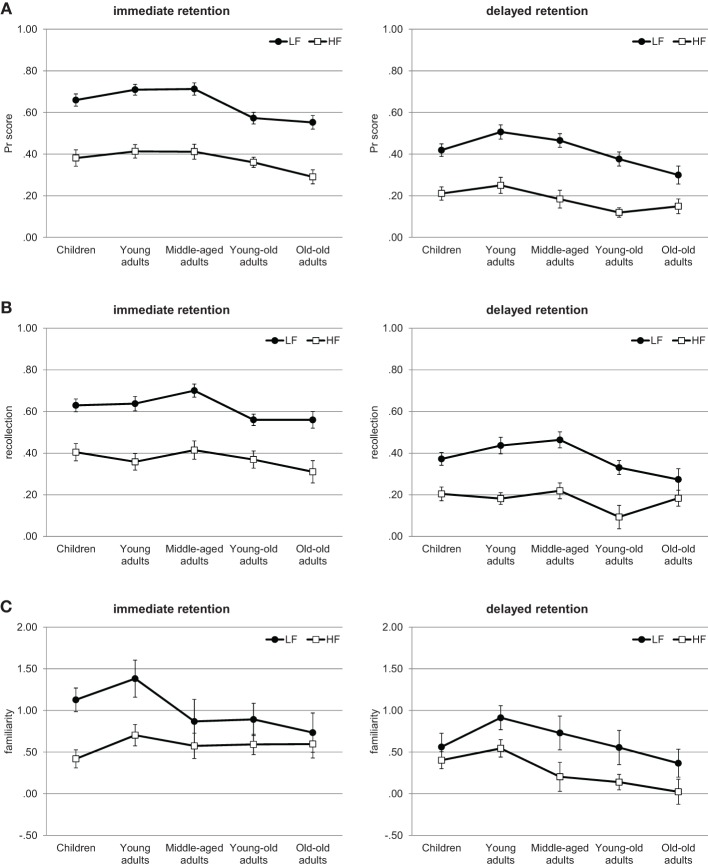
**Experiment 2**. Recognition memory performance across the lifespan. **(A)** *Pr* scores. **(B)** Estimates of recollection. **(C)** Estimates of familiarity. Error bars represent standard errors. LF, low-frequency words; HF, high-frequency words.

For *Pr*, depicted in Figure 2A, a Three-Way ANOVA revealed significant main effects of age, *F*_(4, 137)_ = 5.27, *p* < 0.01, η^2^ = 0.13, retention interval, *F*_(1, 137)_ = 353.1, *p* < 0.001, η^2^ = 0.72, and word-frequency, *F*_(1, 137)_ = 450.61, *p* < 0.001, η^2^ = 0.77. No interaction involving age reached significance, all *F*s < 1.65, *p*s > 0.17. However, the interaction between word frequency and retention interval reached significance, *F*_(1, 137)_ = 3.95, *p* < 0.05, η^2^ = 0.03. Recognition memory performance was higher for low- than for high-frequency words and this difference was larger for the immediate than for the delayed test (immediate: *M* = 0.65 and *M* = 0.38, respectively; delayed: *M* = 0.42 and *M* = 0.19, respectively). *Post-hoc* Tukey HSD tests revealed that the age effect was due the lower performance of the old-old adults compared to the children, the young and the middle-aged adults (*p*s < 0.09 and 0.05, respectively); moreover, the young-old adults performed lower than the young adults (*p* < 0.05). No other group difference reached significance (all *p*s > 0.46).

#### Estimates of recollection

For the estimates of recollection, depicted in Figure 2B, the Three-Way ANOVA showed significant main effects of age, *F*_(4, 137)_ = 2.78, *p* < 0.05, η^2^ = 0.08, retention interval, *F*_(1, 137)_ = 212.05, *p* < 0.001, η^2^ = 0.61, and word frequency, *F*_(1, 137)_ = 299.6, *p* < 0.001, η^2^ = 0.69. There was also a significant interaction between word frequency and retention interval, *F*_(1, 137)_ = 4.12, *p* < 0.05, η^2^ = 0.03. Although recollection was higher for low- than for high-frequency words, this difference was larger for the immediate than for the delayed test (immediate: *M* = 0.62 and *M* = 0.37, respectively; delayed: *M* = 0.38 and *M* = 0.18, respectively). The interaction between age and word frequency was marginally significant, *F*_(4, 137)_ = 2.18, *p* = 0.074, η^2^ = 0.06, but no other interaction approached significance, *F*s < 1.86, *p*s > 0.12. Separate ANOVAs were used to follow-up the interaction between age and word frequency, and showed no effects due to age for high-frequency words, *F*_(4, 137)_ = 1.19, *p* = 0.32, η^2^ = 0.03, together with a significant age effect for low-frequency words, *F*_(4, 137)_ = 4.29, *p* < 0.01, η^2^ = 0.11. *Post-hoc* Tukey HSD tests for low-frequency words revealed that the old-old adults showed less recollection than the young and the middle-aged adults (*p* < 0.08 and 0.05, respectively); moreover, the young-old adults showed less recollection than the middle-aged adults (*p* < 0.05). No other group differences were significant (all *p*s > 0.24).

#### Estimates of familiarity

For the estimates of familiarity, depicted in Figure 2C, the Three-Way ANOVA showed a marginally significant effect of age, *F*_(4, 137)_ = 2.41, *p* = 0.052, η^2^ = 0.07, and significant main effects of retention interval, *F*_(1, 137)_ = 25.64, *p* < 0.001, η^2^ = 0.16, and word-frequency, *F*_(1, 137)_ = 27.39, *p* < 0.001, η^2^ = 0.17. No interaction approached significance, all *F*s < 1.93, *p*s > 0.11. Overall, familiarity was higher for the immediate than delayed test (*M* = 0.79 and *M* = 0.46, respectively) and for low- than for high-frequency words (*M* = 0.83 and *M* = 0.43, respectively). *Post-hoc* Tukey HSD tests revealed that the age effect was due the lower familiarity scores of the old-old adults compared to the young adults (*M* = 0.43 and *M* = 0.89, respectively, *p* < 0.05). No other group difference reached significance (all *p*s > 0.17).

#### Correlation analysis

With the variation of the retention interval within subjects, it was possible to investigate the stability of the estimates of recollection and familiarity. Previous findings have shown that the test-retest reliability of explicit memory tests—assumed to be based on recollection—is higher than the test-retest reliability of implicit memory—assumed to reflect processes such as familiarity (Meier and Perrig, 2000). Accordingly, we expected that the estimates of recollection would more stable across test conditions than those for familiarity. To test this assumption, we computed the correlation between the immediate and delayed tests for each estimate (recollection and familiarity), averaged across word frequencies and age groups. The results revealed significant correlation coefficients for both estimates (recollection: *r* = 0.53; and familiarity: *r* = 0.36, both *p*s < 0.001). More critically, however, a test for correlated but non-overlapping correlations (Raghunathan et al., 1996) showed that these correlations were significantly different, *ZPF* = 1.76, *p* < 0.05. Thus, the estimate of recollection was more stable than the estimate of familiarity and this difference may also have contributed to the presence of a strong age effect for estimates of recollection and a much weaker effect for the estimates of familiarity.

### Discussion

In general, Experiment 2 replicated the results of Experiment 1. As expected, recognition memory performance formed an inverted U-shape function, with higher memory performance in the immediate compared to the delayed test, and also higher performance for low- compared to high-frequency words. Overall, performance appeared to be somewhat lower than in Experiment 1. This is probably due to doubling the number of stimuli, which was a consequence of varying retention interval within-subject. As a result of the within-subject manipulation there was increased power to detect effects of retention interval which appeared in the form of an interaction with word-frequency. However, there was no interaction between retention interval and age. Thus, our hypothesis that an interaction between the retention interval and age may have been disguised by the between-subject manipulation of this variable in Experiment 1 was not confirmed.

For estimates of recollection, there was a marginally significant interaction between age group and word frequency and the pattern of the trajectory was similar to Experiment 1 that is, an age-effect occurred only for low-frequency words, based on the lower estimates of older adults, but no age-effect occurred for high-frequency words. As expected, for estimates of familiarity, there was no significant age effect. However, familiarity was again affected by the retention interval and word-frequency manipulations, with lower estimates for delayed testing and for high-frequency words, thus replicating Experiment 1.

Overall, Experiment 2 corroborates the results of Experiment 1. First, it showed again that for high-frequency words, estimates of recollection did not vary with age. Second, there was still a lack of interaction effects, in particular with age—revealing the substantial stability of recognition memory and its basic processes. Moreover, correlational analysis revealed that the stability of estimates of familiarity was significantly lower than the stability of estimates of recollection and thus may have contributed to the different pattern of age-related trajectories. In Experiment 3, we followed up on this question by introducing a slight variation into the remember/know procedure to enhance the measurement property of the estimates of familiarity. Specifically, we provided an additional “guess” response option (cf. Gardiner et al., 2002). Previous research has shown that when a “guess” response option is available the discrimination between old and new items for “know” responses is increased and thus, the estimates of familiarity may also be improved (Eldridge et al., 2002; cf. Bruno and Rutherford, 2010). Moreover, by eliminating the error variance specific to guess-responses, the stability of the familiarity estimates may be further enhanced. Experiment 3 was similar to Experiment 2, except that we included a “guess” response option in the remember/know procedure.

## Experiment 3

### Method

#### Participants

A total of 192 participants, 39 children, 38 young adults, 40 middle-aged adults, 39 young-old adults, and 36 old-old adults participated in the study. Three of the young-old adults and one of the old-old adults had to be excluded due to a MMS value below 27 points. The data of two participants (one young-old adult and one old-old adult) were lost due to a technical error, thus the final sample consisted of 186 participants. Demographic characteristics of the final sample are presented in Table 1.

#### Materials

Materials were the same as in Experiment 2.

#### Procedure

The procedure was as described in the General Method section. After the 20-min retention interval, the immediate test phase was administered (immediate test). After a 24-h retention interval, the delayed test phase was accomplished in a second session. The only difference from Experiment 2 was that after entering an “old” decision, participants were asked to make Remember, Know, or Guess judgment rather than only a Remember/Know judgment. Specifically, the participants were instructed to make a Remember response when they were able to recollect the word from the study episode, to give a Know response when they were not able to recollect the word, but nevertheless believed that they had studied it before, and to give a Guess response when they only guessed that they had studied it before.

### Results

**Figure 3:**
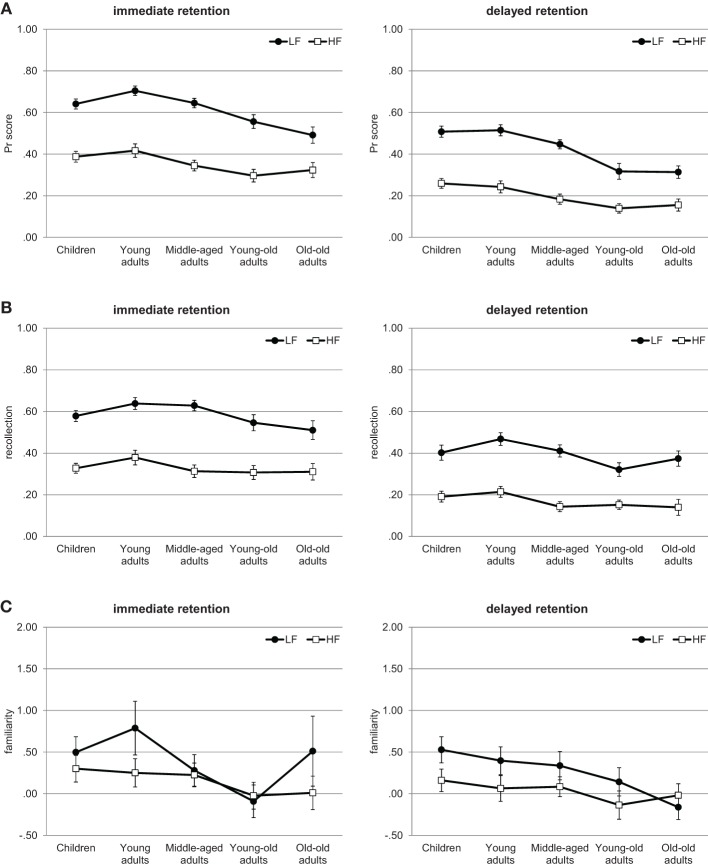
**Experiment 3**. Recognition memory performance across the lifespan. **(A)** *Pr* scores. **(B)** Estimates of recollection. **(C)** Estimates of familiarity. Error bars represent standard errors. LF, low-frequency words; HF, high-frequency words.

Hits and False Alarm rates are presented in Table 4 and the respective analyses are presented in the Supplementary Materials. They showed a consistent word-frequency effect that was slightly smaller in older adults. As in the previous experiments, we focus on recognition memory performance *Pr*, and on the estimates of recollection and on familiarity, which are depicted in Figure 3.

**Table 4:** **Experiment 3: Means and standard errors of hits and false alarm rates**.

		**Children**				**Young adults**				**Middle-aged adults**				**Young-old adults**				**Old-old adults**			
		**Immediate**		**Delayed**		**Immediate**		**Delayed**		**Immediate**		**Delayed**		**Immediate**		**Delayed**		**Immediate**		**Delayed**	
		***M***	***SE***	***M***	***SE***	***M***	***SE***	***M***	***SE***	***M***	***SE***	***M***	***SE***	***M***	***SE***	***M***	***SE***	***M***	***SE***	***M***	***SE***
**REMEMBER**																					
HIT																					
	LF	0.59	0.03	0.43	0.04	0.66	0.03	0.50	0.03	0.64	0.02	0.45	0.03	0.57	0.04	0.39	0.03	0.56	0.04	0.47	0.04
	HF	0.38	0.02	0.23	0.03	0.45	0.03	0.32	0.03	0.38	0.03	0.26	0.03	0.36	0.03	0.24	0.03	0.40	0.04	0.35	0.04
FA																					
	LF	0.04	0.01	0.05	0.01	0.05	0.01	0.06	0.01	0.04	0.01	0.09	0.02	0.07	0.01	0.11	0.02	0.10	0.02	0.17	0.03
	HF	0.08	0.02	0.07	0.02	0.11	0.02	0.13	0.02	0.10	0.02	0.14	0.02	0.09	0.02	0.12	0.02	0.13	0.03	0.23	0.04
**KNOW**																					
HIT																					
	LF	0.12	0.02	0.16	0.02	0.11	0.02	0.11	0.02	0.08	0.01	0.14	0.02	0.06	0.01	0.10	0.02	0.07	0.01	0.10	0.02
	HF	0.15	0.02	0.19	0.02	0.14	0.02	0.17	0.02	0.14	0.02	0.19	0.03	0.10	0.02	0.16	0.03	0.13	0.02	0.14	0.02
FA																					
	LF	0.05	0.01	0.06	0.01	0.04	0.01	0.05	0.01	0.05	0.01	0.07	0.01	0.03	0.01	0.07	0.02	0.05	0.01	0.10	0.02
	HF	0.07	0.01	0.12	0.02	0.11	0.02	0.13	0.02	0.10	0.02	0.14	0.02	0.08	0.02	0.14	0.03	0.09	0.02	0.12	0.02
**GUESS**																					
HIT																					
	LF	0.04	0.01	0.05	0.01	0.05	0.01	0.06	0.01	0.04	0.01	0.05	0.01	0.06	0.01	0.07	0.02	0.06	0.01	0.06	0.02
	HF	0.07	0.02	0.09	0.02	0.10	0.02	0.11	0.02	0.09	0.01	0.09	0.02	0.09	0.02	0.11	0.02	0.09	0.02	0.08	0.02
FA																					
	LF	0.03	0.01	0.03	0.01	0.02	0.00	0.04	0.01	0.02	0.01	0.04	0.01	0.03	0.01	0.06	0.01	0.04	0.01	0.05	0.01
	HF	0.06	0.01	0.07	0.02	0.06	0.01	0.09	0.01	0.07	0.01	0.08	0.01	0.09	0.02	0.11	0.02	0.07	0.02	0.07	0.01
**TOTAL**																					
HIT																					
	LF	0.75	0.02	0.64	0.03	0.82	0.02	0.67	0.02	0.76	0.02	0.65	0.02	0.69	0.03	0.56	0.03	0.68	0.04	0.63	0.04
	HF	0.59	0.03	0.51	0.03	0.69	0.03	0.60	0.03	0.61	0.03	0.54	0.03	0.56	0.04	0.51	0.03	0.61	0.04	0.57	0.04
FA																					
	LF	0.11	0.02	0.13	0.01	0.11	0.01	0.16	0.02	0.12	0.02	0.21	0.02	0.14	0.02	0.24	0.03	0.19	0.03	0.32	0.03
	HF	0.21	0.03	0.25	0.03	0.27	0.03	0.35	0.04	0.26	0.03	0.36	0.03	0.26	0.03	0.38	0.04	0.29	0.04	0.42	0.04

M, mean; SE, standard errors; FA, false alarms; LF, low-frequency words; and HF, high-frequency words.

#### Recognition memory performance

The Three-Way ANOVA of the *Pr* scores, depicted in Figure 3A, revealed significant main effects of age, *F*_(4, 181)_ = 10.49, *p* < 0.001, η^2^ = 0.19, retention interval, *F*_(1, 181)_ = 280.47, *p* < 0.001, η^2^ = 0.61, and word frequency, *F*_(1, 181)_ = 508.68, *p* < 0.001, η^2^ = 0.74. There was also a marginally significant interaction between retention interval and word frequency, *F*_(1, 181)_ = 2.96, *p* = 0.089, η^2^ = 0.02, revealing that although recognition memory performance was higher for low- than for high-frequency words, this difference was larger for the immediate than for the delayed test (immediate: *M* = 0.61 and *M* = 0.36, respectively; delayed: *M* = 0.42 and *M* = 0.20, respectively). More importantly, the interaction between age and word-frequency was significant, *F*_(4, 181)_ = 4.26, *p* < 0.01, η^2^ = 0.09. No other interaction approached significance, all *F*s < 1.22, *p*s > 0.30. Separate ANOVAs into the interaction between age and word frequency showed a significant age effect for high-frequency words, *F*_(4, 181)_ = 4.51, *p* < 0.01, η^2^ = 0.09, as well as for low-frequency words, *F*_(4, 181)_ = 13.03, *p* < 0.001, η^2^ = 0.22. For high-frequency words, *post-hoc* Tukey HSD tests revealed that the age effect was due the lower performance of the young-old and old-old adults compared to the children and the young adults (*p*s < 0.05 and 0.09, respectively). For low-frequency words, *post-hoc* Tukey HSD tests revealed that the age effect was due the lower performance of the young-old and old-old adults compared to the children, the young and the middle-aged adults (all *p*s < 0.05). No other group difference reached significance (all *p*s > 0.34).

#### Estimates of recollection

The estimates of recollection, depicted in Figure 3B, were also examined by a Three-Way ANOVA, which showed significant main effects of age, *F*_(4, 181)_ = 2.65, *p* < 0.05, η^2^ = 0.05, retention interval, *F*_(1, 181)_ = 228.58, *p* < 0.001, η^2^ = 0.56, and word frequency, *F*_(1, 181)_ = 380.5, *p* < 0.001, η^2^ = 0.68. No interaction approached significance, *F*s < 1.91, *p*s > 0.17. Overall, recollection was higher for the immediate than delayed test (*M* = 0.46 and *M* = 0.28, respectively) and for low- than for high-frequency words (*M* = 0.49 and *M* = 0.25, respectively). *Post-hoc* Tukey HSD tests revealed that the age effect was due to the lower recollection of the young-old and old-old adults compared to the young adults (*p*s < 0.05 and 0.06, respectively). No other group difference reached significance (all *p*s > 0.50). Although the interaction between age and word frequency did not reach significance, *F*_(4, 181)_ = 1.63, *p* = 0.17, η^2^ = 0.03, but due to the results of Experiments 1 and 2, we also conducted separate ANOVAs for each frequency condition. Replicating the results of Experiments 1 and 2, there was no age effect for high-frequency words, *F*_(4, 181)_ = 1.52, *p* = 0.20, η^2^ = 0.03. In contrast, for low-frequency words, the age effect was significant, *F*_(4, 181)_ = 3.02, *p* < 0.05, η^2^ = 0.06. *Post-hoc* Tukey HSD tests revealed that the age effect was due the lower recollection of the young-old and old-old adults compared to the young adults (*p*s < 0.05 and 0.06, respectively). No other group difference reached significance (all *p*s > 0.22). Thus, it seems that the lack of significance of the interaction between age and word-frequency is rather due to a lack of statistical power than reflecting a failure to find the data pattern.

#### Estimates of familiarity

For the estimates of familiarity, depicted in Figure 3C, the Three-Way ANOVA showed no significant age effect, *F*_(4, 181)_ = 1.51, *p* = 0.20, η^2^ = 0.03, but a marginally significant effect of retention interval, *F*_(1, 181)_ = 3.74, *p* = 0.055, η^2^ = 0.02, and a significant effect of word frequency, *F*_(1, 181)_ = 12.36, *p* < 0.01, η^2^ = 0.06. No interaction approached significance, all *F*s < 1.75, *p*s > 0.14. Overall, familiarity was higher for the immediate than delayed test (*M* = 0.28 and *M* = 0.15, respectively) and for low- than for high-frequency words (*M* = 0.33 and *M* = 0.10, respectively).

#### Correlation analysis

As in Experiment 2, we investigated the stability of the estimates of recollection and familiarity. Specifically, we were interested whether by the exclusion of guess responses from the calculation of the familiarity estimates the error related variance may be reduced and thus abolishing the reliability differences observed in Experiment 2. Again these scores were significantly different from zero with *r* = 0.56, *p* < 0.01, for recollection *r* = 0.57, *p* < 0.01, for familiarity. More critically, the test for correlated but non-overlapping correlations by Raghunathan et al. (1996) showed no significance, *ZPF* = −0.08, *p* > 0.05. Therefore, the modified remember/know/guess procedure revealed a higher stability across the retention interval for the estimate of familiarity and it eliminated the differences that were present in Experiment 2.

### Discussion

In general, Experiment 3 replicated the main findings from the previous experiments. That is, for low-frequency words, there was a clear inverse u-shape pattern for the age trajectory and a substantial performance decline in old age. However, for high-frequency words, this was less pronounced. For estimates of recollection, there was again a somewhat different pattern for high-and low-frequency words, with significant age-related differences for low-, but not for high-frequency words. In contrast, estimates of familiarity were largely invariable across age, but were again affected by word-frequency, and marginally by the retention interval manipulation. It is noteworthy that the latter results were based on estimates of familiarity that showed comparable stability to the estimate of recollection across the retention interval and thus we can reject the hypothesis that the absence of a significant age-difference in estimates of familiarity is simply a methodological artifact.

## General discussion

The purpose of this study was to investigate the lifespan trajectory of recognition memory test performance, and especially the distinct influences due to recollection and familiarity. We used the same recognition task to examine performance across a large segment of the lifespan that is, five groups ranging from 14 up to 85 years of age. In three separate experiments, we investigated the impact of word-frequency and of different retention intervals, two critical variables known to have different effects on recollection and familiarity. Here, we first summarize the results of the three experiments and then we discuss an additional “meta-analysis” in which we combined the findings from three experiments. We then connect our findings with theoretical approaches of lifespan development and conclude with a more general note.

For recognition memory performance, we found consistent age effects across experiments, with a performance increase from children to young adults and a decline thereafter, thus confirming our hypothesis to find an inverse u-shape lifespan trajectory. As expected, performance was higher when tested on the same day compared to after a 24 h retention interval and for low- compared to high frequency words. Importantly, we found no significant interaction between age group and any experimental variable, thus suggesting a very robust recognition memory performance trajectory across the lifespan. For estimates of recollection the overall pattern was very similar. However, in Experiments 1 and 2, the inverse u-shape lifespan trajectory was only significant for low-frequency words that is, this pattern flattened out for high-frequency words. As hypothesized there was no triple interaction between age, delay, and word-frequency. For estimates of familiarity we did not find a significant age effect. However, estimates of familiarity for the same day were higher compared to after a 24 h retention interval and for low- compared to high-frequency words. Thus, the manipulations of word-frequency and retention interval showed a similar effect for recognition memory performance and the estimates of recollection and familiarity. In contrast, age affected both recognition memory performance and the estimate of recollection but the estimate of familiarity was mainly age-invariant. These results support the notion that recollection and familiarity recruit different processes which are differently affected by development and aging across the lifespan.

Because the method and design were similar across experiments, it was possible to calculate the same statistics based on the combined data from all three experiments. This “meta-analysis” was used to boost statistical power and for revealing effects that might have been missed in the individual experiments. Details about these analyses are presented in the Supplementary Materials. They revealed two additional significant effects. First, for overall recognition memory performance, the age effect was more pronounced for low- than for high-frequency words. Second, and more important, for estimates of familiarity a main effect of age group emerged, with both groups of older adults showing lower familiarity estimates than the young adults. Thus, when a large enough sample is tested, the results show that familiarity is not completely age-invariant. Rather they suggest that familiarity is also somewhat affected in old age, but much less than recollection.

The latter consideration is consistent with conclusions from aging studies. For example, Prull et al. (2006) stated that aging is associated with a stronger impairment in recollection than in familiarity, by contrast to the claim that aging is accompanied by a decline in recollection and invariance in familiarity. However, the absence of a developmental effect (i.e., between children and young adults) would support the pattern of invariance that was found in previous developmental studies (e.g., Ghetti and Angelini, 2008).

Moreover, the absence of a significant developmental effect is in line with the lifespan approach advocated by Shing and Lindenberger (2011). Specifically, according to this approach the associative component of episodic memory matures earlier in childhood than the strategic component which is reflected by an absence of an age effect in familiarity and the presence of an effect in recollection. In contrast, in old age, the associative component is also decreased resulting in both a decline in recollection and familiarity.

To conclude, we would like to emphasize that our study demonstrates that the pattern of age changes across the lifespan is rather robust across different experimental conditions. This suggests that age-related differences in recognition memory are rather stable across different situations—not only for performance measures, but also for estimates of the underlying processes.

### Conflict of interest statement

The authors declare that the research was conducted in the absence of any commercial or financial relationships that could be construed as a potential conflict of interest.

## References

[B1] Balota D. A. Burgess G. C. Cortese M. J. Adams D. R. (2002). The word-frequency mirror effect in young, old, and early-stage Alzheimer's disease: evidence for two processes in episodic recognition performance. J. Mem. Lang. 46, 199–226 10.1006/jmla.2001.2803

[B2] Baltes P. B. (1987). Theoretical propositions of life-span developmental psychology: on the dynamics between growth and decline. Dev. Psychol. 23, 611–626 10.1037/0012-1649.23.5.61117158023

[B3] Baltes P. B. Lindenberger U. Staudinger U. M. (2006). Lifespan theory in developmental psychology, in Handbook of Child Psychology: Theoretical Models of Human Development, 6th edn. eds Damon W. Lerner R. M. (New York, NY: Wiley), 569–664

[B4] Bastin C. Van der Linden M. (2003). The contribution of recollection and familiarity to recognition memory: a study of the effects of test format and aging. Neuropsychology 17, 14–24 10.1037/0894-4105.17.1.14 12597069

[B5] Billingsley R. L. Smith M. McAndrews M. (2002). Developmental patterns in priming and familiarity in explicit recollection. J. Exp. Child Psychol. 82, 251–277 10.1016/S0022-0965(02)00007-3 12093109

[B6] Bruno D. Rutherford A. (2010). How many response options? A study of remember–know testing procedures. Acta Psychol. 134, 125–129 10.1016/j.actpsy.2010.01.002 20137771

[B7] Bugaiska A. Clarys D. Jarry C. Taconnat L. Tapia G. Vanneste S. (2007). The effect of ageing in recollective experience: the processing speed and executive functioning hypothesis. Conscious. Cogn. 16, 797–808 10.1016/j.concog.2006.11.007 17251040

[B8] Bunce D. (2003). Cognitive support at encoding attenuates age differences in recollective experience among adults of lower frontal lobe function. Neuropsychology 17, 353–361 10.1037/0894-4105.17.3.353 12959501

[B9] Bunce D. Macready A. (2005). Processing speed, executive function, and age differences in remembering and knowing. The Q. J. Exp. Psychol. 58, 155–168 10.1080/02724980443000197 15881296

[B10] Clarys D. Isingrini M. Gana K. (2002). Ageing and episodic memory: mediators of age differences in remembering and knowing. Acta Psychol. 109, 315–329 10.1016/S0001-6918(01)00064-6 11881906

[B11] Comblain C. D'Argembeau A. Van Der Linden M. Aldenhoff L. (2004). The effect of ageing on the recollection of emotional and neutral picture. Memory 12, 673–684 10.1080/09658210344000477 15724356

[B12] Craik F. I. M. Bialystok E. (2006). Cognition through the lifespan cognition: mechanisms of change. Trends Cogn. Sci. 10, 131–138 10.1016/j.tics.2006.01.007 16460992

[B13] Craik F. I. M. McDowd J. M. (1987). Age differences in recall and recognition. J. Exp. Psychol. Learn. Mem. Cogn. 13, 474–479 10.1037/0278-7393.13.3.47416460992

[B14] Duarte A. Henson R. N. Graham K. S. (2008). The effects of aging on the neural correlates of subjective and objective recollection. Cereb. Cortex 18, 2169–2180 10.1093/cercor/bhm243 18165281 PMC2517104

[B15] Duarte A. Ranganath C. Trujillo C. Knight R. T. (2006). Intact recollection memory in high-performing older adults: ERP and behavioral evidence. J. Cogn. Neurosci. 18, 33–47 10.1162/089892906775249988 16417681

[B16] Eldridge L. L. Sarfatti S. Knowlton B. J. (2002). The effect of testing procedure on remember–know judgments. Psychon. Bull. Rev. 9, 139–145 10.3758/BF03196270 12026946

[B17] Fingerman K. L. Berg C. A. Smith J. Antonucci T. C. (2011). Handbook of the Lifespan Development. New York, NY: Springer Publishing Company

[B18] Folstein M. F. Folstein S. E. McHugh P. R. (1975). Mini-mental state: a practical method for grading the cognitive state of patients for the clinician. J. Psychiatr. Res. 12, 189–198 10.1016/0022-3956(75)90026-6 1202204

[B19] Friedman D. de Chastelaine M. Nessler D. Malcolm B. (2010). Changes in familiarity and recollection across the lifespan: an ERP perspective. Brain Res. 1310, 124–141 10.1016/j.brainres.2009.11.016 19914220 PMC2812671

[B20] Friedman D. Trott C. (2000). An event-related potential study of encoding in young and older adults. Neuropsychologia 38, 542–557 10.1016/S0028-3932(99)00122-0 10689032

[B21] Gardiner J. M. Java R. I. (1990). Recollective experience in word and nonword recognition. Mem. Cogn. 18, 23–30 10.3758/BF03202642 2314224

[B22] Gardiner J. M. Java R. I. (1991). Forgetting in recognition memory with and without recollective experience. Mem. Cogn. 19, 617–623 10.3758/BF03197157 1758306

[B23] Gardiner J. M. Ramponi C. Richardson-Klavehn A. (2002). Recognition memory and decision processes: a meta-analysis of remember, know, and guess responses. Memory 10, 83–98 10.1080/09658210143000281 11798439

[B24] Gardiner J. M. Richardson-Klavehn A. Ramponi C. (1997). On reporting recollective experiences and “direct access to memory systems.” Psychol. Sci. 8, 391–394 10.1111/j.1467-9280.1997.tb00431.x

[B25] Ghetti S. Angelini L. (2008). The development of recollection and familiarity in childhood and adolescence: evidence from dual process signal detection. Child Dev. 79, 339–358 10.1111/j.1467-8624.2007.01129.x 18366427

[B26] Glanzer M. Adams J. K. (1985). The mirror effect in recognition memory. Mem. Cogn. 13, 8–20 10.3758/BF03198438 4010518

[B27] Glanzer M. Adams J. K. (1990). The mirror effect in recognition memory: data and theory. J. Exp. Psychol. Learn. Mem. Cogn. 16, 5–16 10.1037/0278-7393.16.1.5 2136752

[B28] Graf P. Ohta N. (2002). Lifespan Memory Development. Cambridge, MA: MIT Press

[B29] Gregg V. H. Montgomery D. C. Castano D. (1980). Recall of common and uncommon words from pure and mixed lists. J. Verbal Learn. Verbal Behav. 19, 240–245 10.1016/S0022-5371(80)90202-9

[B30] Guttentag R. E. Carroll D. (1997). Recollection-based recognition: word frequency effects. J. Mem. Lang. 37, 502–516 10.1006/jmla.1997.2532

[B31] Hintzman D. L. Caulton D. A. (1997). Recognition memory and modality judgments: a comparison of retrieval dynamics. J. Mem. Lang. 37, 1–23 10.1006/jmla.1997.2511

[B32] Hirshman E. Fisher J. Henthorn T. Arndt J. Passannante A. (2002). Midazolam amnesia and dual-process models of the word-frequency mirror effect. J. Mem. Lang. 47, 499–516 10.1016/S0749-596X(02)00017-7

[B33] Hockley W. E. (1991). Recognition memory for item and associative information: a comparison of forgetting rates, in Relating Theory and Data: Essays on Human Memory in Honor of Bennet B. Murdock, eds William E. Hockley E. Lewandowsky E. S. (Hillsdale, NJ: Erlbaum), 227–248

[B34] Hockley W. E. (1992). Item versus associative information: further comparisons of forgetting rates. J. Exp. Psychol. Learn. Mem. Cogn. 18, 1321–1330 10.1037/0278-7393.18.6.1321

[B35] Hockley W. E. Consoli A. (1999). Familiarity and recollection in item and associative recognition. Mem. Cogn. 27, 657–664 10.3758/BF03211559 10479824

[B36] Java R. I. (1996). Effects of age on state of awareness following implicit and explicit word-association tasks. Psychol. Aging 11, 108–111 10.1037/0882-7974.11.1.108 8726376

[B37] Joordens S. Hockley W. E. (2000). Recollection and familiarity through the looking glass: when old does not mirror new. J. Exp. Psychol. Learn. Mem. Cogn. 26, 1534–1555 10.1037/0278-7393.26.6.1534 11185781

[B38] Kinoshita S. (1995). The word frequency effect in recognition memory versus repetition priming. Mem. Cogn. 23, 569–580 10.3758/BF03197259 7476243

[B39] Lehrl S. Merz J. Burkhard G. Fischer B. (1991). MWT-A: Mehrfachwahl-Wortschatz-Intelligenztest. Parallelform zum MWTB. Erlangen: Perimed

[B40] Li S.-C. Baltes P. B. (2006). Cognitive developmental research from lifespan perspectives: the challenge of integration, in Lifespan Cognition: Mechanisms of Change, eds Bialystok E. Craik F. I. M. (New York, NY: Oxford University press), 344–363 10.1093/acprof:oso/9780195169539.003.0024

[B41] Light L. L. Prull M. W. Lavoie D. J. Healy M. R. (2000). Dual process theories of memory in old age, in Models of Cognitive Ageing, eds Perfect T. J. Maylor E. A. (Oxford, UK: Oxford University Press), 238–300

[B42] Lövdén M. Rönnlund M. Nilson G.-L. (2002). Remembering and knowing in adulthood: effects of enacted encoding and relations to processing speed. Aging Neuropsychol. Cogn. 9, 184–200 10.1076/anec.9.3.184.9612

[B43] Mandler G. (1979). Organization and repetition: organizational principles with special reference to rote learning, in Perspectives on Memory Research, ed Nilsson L. G. (Hillsdale, NJ: Erlbaum), 293–327

[B44] Mäntylä T. (1993). Knowing but not remembering: adult age differences in recollective experience. Mem. Cogn. 21, 379–388 10.3758/BF03208271 8316101

[B45] Mark R. E. Rugg M. D. (1998). Age effects on brain activity associated with episodic memory retrieval: an electrophysiological study. Brain 121, 861–873 10.1093/brain/121.5.861 9619190

[B46] Mecklinger A. Brunnemann N. Kipp K. (2010). Two processes for recognition memory in children of early school age: an event-related study. J. Cogn. Neurosci. 23, 435–446 10.1162/jocn.2010.21455 20146611

[B47] Meier B. Perrig W. J. (2000). Low reliability of perceptual priming: consequences for the interpretation of functional dissociations between explicit and implicit memory. Q. J. Exp. Psychol. A Hum. Exp. Psychol. 53, 211–233 10.1080/027249800390745 10718071

[B48] Meier B. Perrig-Chiello P. Perrig W. (2002). Personality and memory in old age. Aging Neuropsychol. Cogn. 9, 135–144 10.1076/anec.9.2.135.9544

[B49] Meier B. Theiler-Bürgi M. Perrig W. (2009). Levels of processing and amnesia affect perceptual priming in fragmented picture naming. Int. J. Neurosci. 119, 1061–1075 10.1080/00207450802336691 19922339

[B50] Norman K. A. Schacter D. L. (1997). False recognition in younger and older adults: exploring the characteristics of illusory memories. Mem. Cogn. 25, 838–848 10.3758/BF03211328 9421570

[B51] Ofen N. Kao Y. C. Sokol-Hessner P. Kim H. Whitfield-Gabrieli S. Gabrieli J. D. (2007). Development of the declarative memory system in the human brain. Nat. Neurosci. 10, 1198–1205 10.1038/nn1950 17676059

[B52] Parkin A. J. Walter B. M. (1992). Recollective experience, normal aging, and frontal dysfunction. Psychol. Aging 7, 290–298 10.1037/0882-7974.7.2.290 1610518

[B53] Perfect T. J. Dasgupta Z. R. R. (1997). What underlies the deficit in reported recollective experience in old age. Mem. Cogn. 25, 849–858 10.3758/BF03211329 9421571

[B54] Perfect T. J. Williams R. B. Anderton-Brown C. (1995). Age differences in reported recollective experiences are due to encoding effects, not response bias. Memory 3, 169–184 10.1080/09658219508258964 7796303

[B55] Prull M. W. Dawes C. L. L. Martin A. M. Rosenberg H. F. Light L. L. (2006). Recollection and familiarity in recognition memory: adult age differences and neuropsychological test correlates. Psychol. Aging 21, 107–118 10.1037/0882-7974.21.1.107 16594796

[B56] Raghunathan T. E. Rosenthal R. Rubin D. B. (1996). Comparing correlated but nonoverlapping correlations. Psychol. Methods 1, 178–183 10.1037/1082-989X.1.2.178

[B57] Reder L. M. Nhouyvanisvong A. Schunn C. D. Ayers M. S. Angstadt P. Hiraki K. (2000). A mechanistic account of the mirror effect for word frequency: a computational model of remember-know judgments in a continuous recognition paradigm. J. Exp. Psychol. Learn. Mem. Cogn. 26, 294–320 10.1037/0278-7393.26.2.294 10764098

[B58] Salthouse T. A. (1985). Speed of behavior and its implications for cognition, in Handbook of the Psychology of Aging, eds Birren J. E. Schaie K. W. (New York, NY: Van Nostrand Reinhold), 400–426

[B59] Salthouse T. A. (1996). The processing-speed theory of adult age differences in cognition. Psychol. Rev. 103, 403–428 10.1037/0033-295X.103.3.403 8759042

[B60] Schacter D. L. Koutstaal W. Johnson M. K. Gross M. S. Angell K. E. (1997). False recollection induced by photographs: a comparison of older and younger adults. Psychol. Aging 12, 203–215 10.1037/0882-7974.12.2.203 9189980

[B61] Shing Y. L. Lindenberger U. (2011). The development of episodic memory: lifespan lessons. Child Dev. Perspect. 5, 148–155 10.1111/j.1750-8606.2011.00170.x

[B62] Shing Y. L. Werkle-Bergner M. Li S.-C. Lindenberger U. (2008). Associative and strategic components of episodic memory: a life span dissociation. J. Exp. Psychol. Gen. 137, 495–513 10.1037/0096-3445.137.3.495 18729712

[B63] Skinner E. I. Fernandes M. A. (2009). Effect of study context on item recollection. Q. J. Exp. Psychol. 63, 1318–1334 10.1080/17470210903348613 19890765

[B64] Snodgrass J. G. Corwin J. (1988). Pragmatics of measuring recognition memory: applications to dementia and amnesia. J. Exp. Psychol. Gen. 117, 34–50 10.1037/0096-3445.117.1.34 2966230

[B65] Tulving E. (1985). Memory and consciousness. Can. Psychol. 26, 1–12 10.1037/h0080017

[B66] Tulving E. (2002). Episodic memory: from mind to brain. Ann. Rev. Psychol. 53, 1–25 10.1146/annurev.psych.53.100901.135114 11752477

[B67] Weiermann B. Stephan M. A. Kaelin-Lang A. Meier B. (2010). Is there a recognition memory deficit in Parkinson's Disease? Evidence from estimates of recollection and familiarity. Int. J. Neurosci. 120, 211–216 10.3109/00207450903506510 20374089

[B68] Wilson R. S. Bacon L. D. Fox J. H. Kramer R. L. Kaszniak A. W. (1983). Word frequency effect and recognition memory in dementia of the Alzheimer type. J. Clin. Neuropsychol. 5, 97–104 10.1080/01688638308401157 6863566

[B69] Yonelinas A. P. (2002). The nature of recollection and familiarity: a review of 30 years of research. J. Mem. Lang. 46, 441–517 10.1006/jmla.2002.286416899208

[B70] Yonelinas A. P. Kroll N. E. A. Dobbins I. Lazzara M. Knight R. T. (1998). Recollection and familiarity deficits in amnesia: convergence of remember-know, process dissociation, and receiver operating characteristic data. Neuropsychology 12, 323–339 10.1037/0894-4105.12.3.323 9673991

