# Supplementary material for: Recognition memory across the lifespan: the impact of word frequency and study-test interval on estimates of familiarity and recollection
Source: Front Psychol. 2013 Oct 30;4:787. doi: 10.3389/fpsyg.2013.00787 (PMC3812907; doi:10.3389/fpsyg.2013.00787)
Supplement: Supplementary file 1 [file DataSheet1.DOCX]

**Supplementary materials**

**Analyses of the word-frequency mirror effect**

1. **Experiment 1**

Overall, hit and false alarm rates showed the expected word-frequency mirror effect. For the hit rates, a three-way analysis of variance (ANOVA) with age group (children, young adults, middle-aged adults, young-old adults, old-old adults) and retention interval (immediate test, delayed test) as between-subjects factors and word-frequency (high, low) as a within-subject factor showed a marginally significant main effect of age, *F* (4, 171) = 2.42, *p* = .05, *η*^2^ = .05, and significant main effects of retention interval, *F* (1, 171) = 12.02, *p* < .01, *η*^2^ = .07, and word frequency, *F* (1, 171) = 69.02, *p* < .001, *η*^2^ = .29. No interaction reached significance, *F*s < 1.78, *p*s > .13. Overall, hits were higher for the immediate than delayed test (*M* = .71 and .63, respectively) and for low- than for high-frequency words (*M* = .73 and .61, respectively). Post-hoc Tukey HSD tests revealed that the age effect was due the lower hits of the children compared to the young adults and lower hits of old-old adults and young adults (*p*s < .05). No other group difference reached significance (all *p*s > .11).

A similar ANOVA of the false alarms showed a marginally significant main effect of age, *F* (4, 171) = 2.24, *p* = .07, *η*^2^ = .05, and significant main effects of retention interval, *F* (1, 171) = 10.07, *p* < .01, *η*^2^ = .06, and word frequency, *F* (1, 171) = 77.56, *p* < .001, *η*^2^ = .31. No interaction reached significance, *F*s < 1.27, *p*s > .28. Overall, false alarms were lower for the immediate than delayed test (*M* = .17 and .24, respectively) and for low- than for high-frequency words (*M* = .16 and .25, respectively). Post-hoc Tukey HSD tests revealed no significant group differences (all *p*s > .10). Together, the results of Experiment 1 showed a consistent word-frequency mirror-effect that was present for all age groups.

1. **Experiment 2**

For the hit rates, a three-way ANOVA with age group (children, young adults, middle-aged adults, young-old adults, old-old adults) as a between-subjects factor and retention interval (immediate test, delayed test) and word-frequency (high, low) as within-subject factors showed no age effect, *F* (4, 137) = 1.37, *p* = .25, *η*^2^ = .04, but significant main effects of retention interval, *F* (1, 137) = 96.18, *p* < .001, *η*^2^ = .41, and word frequency, *F* (1, 137) = 80.95, *p* < .001, *η*^2^ = .37, and significant interactions between retention interval and word frequency, *F* (1, 137) = 6.38, *p* < .05, *η*^2^ = .04, as well as between age and word frequency, *F* (4, 137) = 3.16, *p* < .05, *η*^2^ = .08. Hits were higher for low- than for high-frequency words and this difference was larger for the immediate than for the delayed test (immediate: *M* = .76 and .63, respectively; delayed: *M* = .63 and .53, respectively). Separate ANOVAs were used to follow-up the interaction between age and word frequency, and showed no effects due to age for high-frequency words, *F* (4, 137) = 1.08, *p* = .37, *η*^2^ = .03, together with a significant age effect for low-frequency words, *F* (4, 137) = 3.00, *p* < .05, *η*^2^ = .08. Post-hoc Tukey HSD tests for low-frequency words revealed that old-old and young-old adults showed lower hits than the middle-aged adults (*p*s < .05 and .09, respectively). No other group difference reached significance (all *p*s > .14).

A similar ANOVA of the false alarms showed significant main effects of age, *F* (4, 137) = 4.32, *p* < .01, *η2* = .11, retention interval, *F* (1, 137) = 83.14, *p* < .001, *η2* = .38, and word frequency, *F* (1, 137) = 131.45, *p* < .001, *η2* = .49. No interaction reached significance, *Fs* < 2.08, *ps* > .08. Overall, false alarms were lower for the immediate than delayed test (*M* = .19 and .28, respectively) and for low- than for high-frequency words (*M* = .16 and .30, respectively). Post-hoc Tukey HSD tests revealed that the age effect was due the higher false alarms of the old-old adults compared to both the children and the young adults (*p* < .05 and .01, respectively); moreover, young-old adults had higher false alarms than young adults (*p* < .10). No other group difference reached significance (all *p*s > .22).

Taken together, the hit and false alarm rates again revealed a word-frequency mirror effect as in Experiment 1. However, there were differences between the two experiments, which were mainly caused by the higher hit and false alarm rates of the middle-aged adults in Experiment 2.

1. **Experiment 3**

For the hit rates, a three-way ANOVA with age group (children, young adults, middle-aged adults, young-old adults, old-old adults) as a between-subjects factor, and retention interval (immediate test, delayed test) and word-frequency (high, low) as within-subject factors showed significant main effects of age, *F* (4, 181) = 3.26, *p* < .05, *η*^2^ = .07, retention interval, *F* (1, 181) = 65.85, *p* < .001, *η*^2^ = .27, and word frequency, *F* (1, 181) = 85.35, *p* < .001, *η*^2^ = .32, as well as a significant interaction between the latter factors, *F* (1, 181) = 8.52, *p* < .01, *η*^2^ = .04. No other interaction reached significance, *Fs* < 1.67, *ps* > .16. Again, hits were higher for low- than for high-frequency words and this difference was larger for the immediate than for the delayed test (immediate: M = .74 and .61, respectively; delayed: M = .63 and .55, respectively). Post-hoc Tukey HSD tests revealed that the age effect was due the lower hit rate of the young-old adults compared to the young adults (*p* < .01). No other group difference reached significance (all *p*s > .16).

A similar ANOVA of the false alarms showed significant main effects of age, *F* (4, 181) = 4.61, *p* < .01, *η*^2^ = .09, retention interval, *F* (1, 181) = 87.41, *p* < .001, *η*^2^ = .33, and word frequency, *F* (1, 181) = 183.27, *p* < .001, *η*^2^ = .50, together with a significant interaction between age and retention interval, *F* (4, 181) = 3.61, *p* < .01, *η*^2^ = .07, and a marginally significant interaction between age and word-frequency, *F* (4, 181) = 2.17, *p* = .07, *η*^2^ = .05. Follow-up analyses showed a significant age effect in the delayed test, *F* (4, 181) = 6.18, *p* < .001, *η*^2^ = .12, but not in the immediate test, *F* (4, 181) = 1.80, *p* = .13, *η*^2^ = .04. Post-hoc Tukey HSD tests for the delayed test revealed that this age effect was due the higher false alarms of the old-old adults compared to both the children and the young adults (both *p*s < .05); moreover, young-old and middle-aged adults had higher false alarms than the children (*p*s < .05 and .08, respectively). Furthermore, follow-up analyses showed significant age effects for both low- and high-frequency words, *F* (4, 181) = 8.02, *p* < .001, *η*^2^ = .15, and *F* (4, 181) = 2.62, *p* < .05, *η*^2^ = .05. For low-frequency words, post-hoc Tukey HSD tests revealed that the age effect was due the higher false alarms of the old-old adults compared to the children, the young, and the middle-aged adults (all *p*s < .01); moreover, young-old adults had higher false alarms than the children (*p* < .06). For high-frequency words, the age effect was due the higher false alarms of the old-old adults compared to the children (*p* < .05). No other group difference reached significance (all *p*s > .14).

Taken together, hit and false alarm rates again revealed a word-frequency mirror effect. The effect was stronger for immediate than for delayed testing and as in Experiment 2 there was again a tendency for a smaller word-frequency mirror effect in old age (cf. Balota et al., 2002).

1. **Combined analysis of all three experiments**

As all three experiments were very similar, we performed a three-way ANOVA in which the data of all three experiments were included. It is to note that in Experiment 1, retention interval was manipulated between-subjects, while in Experiments 2 and 3, it was manipulated within-subject. To analyze the data from all three experiments in the same analysis, we transformed the retention interval from Experiments 2 and 3 in a between-subjects variable. Therefore, we conducted a three-way ANOVA, with age group (children, young adults, middle-aged adults, young-old adults, old-old adults) and retention interval (immediate test, delayed test) as between-subjects factors and word frequency (high, low) as a within-subject factor. The word-frequency mirror-effect with hits and false alarm rates is presented in the Supplementary Table. The Pr scores and the estimates of recollection and familiarity are depicted in the Supplementary Figure.

**Word-frequency mirror effect**. For the hit rates, a three-way ANOVA with age group (children, young adults, middle-aged adults, young-old adults, old-old adults) and retention interval (immediate test, delayed test) as between-subjects factors and word-frequency (high, low) as a within-subject factor showed significant main effects of age, *F* (4, 827) = 6.08, *p* < .001, *η*^2^ = .03, retention interval, *F* (1, 827) = 75.99, *p* < .001, *η*^2^ = .08, and word frequency, *F* (1, 827) = 302.38, *p* < .001, *η*^2^ = .27. There was also a significant interaction between retention interval and word frequency, *F* (1, 827) = 4.87, *p* < .05, *η*^2^ = .01. Hits were higher for low- than for high-frequency words and this difference was larger for the immediate than for the delayed test (immediate: M = .75 and .63, respectively; delayed: M = .64 and .55, respectively). More importantly, the interaction between age and word frequency was significant, *F* (4, 827) = 4.96, *p* < .01, *η*^2^ = .02. No other interaction reached significance, *Fs* < 1, *ps* > .61. Separate ANOVAs into the interaction between age and word frequency showed a significant age effect for high-frequency words, *F* (4, 837) = 3.24, *p* < .05, *η*^2^ = .01, as well as for low-frequency words, *F* (4, 837) = 8.75, *p* < .001, *η*^2^ = .04. For high-frequency words, post-hoc Tukey HSD tests revealed that the age effect was caused by the lower hits of the children compared to the young adults (*p* < .01). For low-frequency words, the age effect was caused not only by the lower hits of the children compared to the young adults (*p* < .05), but also by the lower hits of the young-old and old-old adults compared to the young and middle-aged adults (all *ps* < .01). No other group difference reached significance (all *p*s > .11).

((Supplementary Table))

A similar three-way ANOVA of the false alarms showed significant main effects of age, *F* (4, 827) = 15.05, *p* < .001, *η*^2^ = .07, retention interval, *F* (1, 827) = 64.75, *p* < .001, *η*^2^ = .07, and word frequency, *F* (1, 827) = 501.12, *p* < .001, *η*^2^ = .38. No interaction reached significance, *Fs* < 1.63, *ps* > .16. Overall, false alarms were lower for the immediate than delayed test (M = .19 and .27, respectively) and for low- than for high-frequency words (M = .17 and .29, respectively). Post-hoc Tukey HSD tests revealed that the age effect was caused by the higher false alarms of the young-old and old-old adults compared to both the children and the young adults (all *ps* < .01); moreover, the middle-aged adults showed higher false alarms than the children (*p* < .05), but lower false alarms than the old-old adults (*p* < .01). No other group difference reached significance (all *p*s > .13).

**Recognition performance**. For the Pr data (Supplementary Figure, Panel A), a similar three-way ANOVA revealed significant main effects of age, *F* (4, 827) = 26.54, *p* < .001, *η*^2^ = .11, retention interval, *F* (1, 827) = 280.27, *p* < .001, *η*^2^ = .25, and word frequency, *F* (1, 827) = 1315.39, *p* < .001, *η*^2^ = .61. There was also a marginally significant interaction between retention interval and word frequency, *F* (1, 827) = 3.58, *p* = .059, *η*^2^ = .004. Recognition memory performance was higher for low- than for high-frequency words and this difference was larger for the immediate than for the delayed test (immediate: M = .63 and .38, respectively; delayed: M = .43 and .21, respectively). More importantly, the interaction between age and word-frequency was significant, *F* (4, 827) = 7.04, *p* < .001, *η*^2^ = .03. Separate ANOVAs into the interaction between age and word frequency showed a significant age effect for high-frequency words, *F* (4, 837) = 8.14, *p* < .001, *η*^2^ = .04, as well as for low-frequency words, *F* (4, 837) = 25.95, *p* < .001, *η*^2^ = .11. For high-frequency words, post-hoc Tukey HSD tests revealed that the age effect was caused by the lower recognition performance of the young-old and old-old adults compared to both the children (*p* < .06 and .05, respectively) and the young adults (both *p*s < .001); moreover, the old-old adults had lower recognition performance than the middle-aged adults (*p* < .08). For low-frequency words, the age effect was caused by the lower recognition performance of the young-old and old-old adults compared to the children, the young adults and the middle-aged adults (all *p*s < .001). No other group difference reached significance (all *p*s > .12).

((Supplementary Figure))

**Recollection**. The estimate of recollection (Supplementary Figure, Panel B) was also examined by a three-way ANOVA, which showed significant main effects of age, *F* (4, 827) = 8.76, *p* < .001, *η*^2^ = .04, retention interval, *F* (1, 827) = 237.17, *p* < .001, *η*^2^ = .22, and word frequency, *F* (1, 827) = 991.66, *p* < .001, *η*^2^ = .54. There was also a significant interaction between retention interval and word frequency, *F* (1, 827) = 4.64, *p* < .05, *η*^2^ = .01. Recollection was higher for low- than for high-frequency words and this difference was larger for the immediate than for the delayed test (immediate: M = .60 and .36, respectively; delayed: M = .40 and .19, respectively). More importantly, the interaction between age and word-frequency was significant, *F* (4, 827) = 5.47, *p* < .001, *η*^2^ = .03. No other interaction approached significance, all *F*s < 1, *p*s > .79. Separate ANOVAs into the interaction between age and word frequency showed a significant age effect for low-frequency words, *F* (4, 837) = 11.09, *p* < .001, *η*^2^ = .05, but not for high-frequency words, *F* (4, 837) = 1.85, *p* = .12, *η*^2^ = .01. Post-hoc Tukey HSD tests for low-frequency words revealed that the age effect was due the lower recollection of the young-old and old-old adults compared to the children, the young adults and the middle-aged adults (*p*s < .001, except for the comparison with the children with *p* < .09 and .05, respectively).

**Familiarity.** For the estimate of familiarity (Supplementary Figure, Panel C), the three-way ANOVA showed significant main effects of age, *F* (4, 827) = 4.26, *p* < .01, *η*^2^ = .02, retention interval, *F* (1, 827) = 15.44, *p* < .001, *η*^2^ = .02, and word frequency, *F* (1, 827) = 47.92, *p* < .001, *η*^2^ = .05. No interaction approached significance, all *F*s < 1.84, *p*s > .12. Overall, familiarity was higher for the immediate than delayed test (M = .57 and .33, respectively) and for low- than for high-frequency words (M = .60 and .31, respectively). Post-hoc Tukey HSD tests revealed that the age effect was due the lower familiarity of the young-old and old-old adults compared to the young adults (all *p*s < .01).

**Pooled analysis of Experiment 2 and 3.** As noted above, in order to combine all three experiments, we treated the within-subjects variable retention interval of Experiment 2 and 3 as a between-subject variable. While this has boosted the N for the analysis, it also may have led to an underestimation of the effects involving retention interval. In order to exclude the possibility that we may have missed such an effect, we also performed additional analyses in which we combined only Experiments 2 and 3, but kept the within-subject manipulation. These analyses showed the same pattern of results and did not reveal any additional interactions. Thus we are confident that we have teased out all the relevant effects in the reported combined analyses.

Supplementary Table

*All 3 experiments: Means and standard errors of hits and false alarm rates*

|  |  |  | Children | | | | Young adults | | | | Middle-aged adults | | | | Young-old adults | | | | Old-old adults | | | |
| --- | --- | --- | --- | --- | --- | --- | --- | --- | --- | --- | --- | --- | --- | --- | --- | --- | --- | --- | --- | --- | --- | --- |
|  |  |  | Immediate | | Delayed | | Immediate | | Delayed | | Immediate | | Delayed | | Immediate | | Delayed | | Immediate | | Delayed | |
|  |  |  | M | SE | M | SE | M | SE | M | SE | M | SE | M | SE | M | SE | M | SE | M | SE | M | SE |
| Remember | |  |  |  |  |  |  |  |  |  |  |  |  |  |  |  |  |  |  |  |  |  |
|  | HIT |  |  |  |  |  |  |  |  |  |  |  |  |  |  |  |  |  |  |  |  |  |
|  |  | LF | .62 | .02 | .45 | .02 | .66 | .02 | .50 | .02 | .68 | .02 | .49 | .02 | .59 | .02 | .42 | .02 | .57 | .03 | .43 | .03 |
|  |  | HF | .40 | .02 | .27 | .02 | .44 | .02 | .29 | .02 | .43 | .02 | .30 | .02 | .41 | .02 | .29 | .03 | .43 | .03 | .32 | .03 |
|  | FA |  |  |  |  |  |  |  |  |  |  |  |  |  |  |  |  |  |  |  |  |  |
|  |  | LF | .04 | .01 | .06 | .01 | .04 | .01 | .05 | .01 | .04 | .01 | .10 | .01 | .07 | .01 | .12 | .01 | .10 | .01 | .15 | .02 |
|  |  | HF | .06 | .01 | .09 | .01 | .08 | .01 | .09 | .01 | .10 | .01 | .13 | .02 | .11 | .02 | .16 | .02 | .15 | .02 | .19 | .02 |
| Know |  |  |  |  |  |  |  |  |  |  |  |  |  |  |  |  |  |  |  |  |  |  |
|  | HIT |  |  |  |  |  |  |  |  |  |  |  |  |  |  |  |  |  |  |  |  |  |
|  |  | LF | .12 | .01 | .16 | .01 | .12 | .01 | .15 | .01 | .09 | .01 | .16 | .01 | .09 | .01 | .16 | .01 | .11 | .01 | .16 | .02 |
|  |  | HF | .15 | .01 | .20 | .01 | .18 | .01 | .23 | .02 | .18 | .02 | .20 | .02 | .15 | .01 | .21 | .02 | .17 | .02 | .20 | .02 |
|  | FA |  |  |  |  |  |  |  |  |  |  |  |  |  |  |  |  |  |  |  |  |  |
|  |  | LF | .05 | .01 | .08 | .01 | .05 | .01 | .08 | .01 | .05 | .01 | .09 | .01 | .05 | .01 | .10 | .01 | .08 | .01 | .12 | .01 |
|  |  | HF | .11 | .01 | .15 | .01 | .14 | .01 | .16 | .01 | .12 | .01 | .18 | .02 | .10 | .01 | .18 | .02 | .13 | .02 | .18 | .02 |
| Total |  |  |  |  |  |  |  |  |  |  |  |  |  |  |  |  |  |  |  |  |  |  |
|  | HIT |  |  |  |  |  |  |  |  |  |  |  |  |  |  |  |  |  |  |  |  |  |
|  |  | LF | .76 | .01 | .63 | .02 | .80 | .01 | .68 | .02 | .79 | .01 | .68 | .02 | .71 | .02 | .60 | .02 | .70 | .02 | .62 | .02 |
|  |  | HF | .58 | .02 | .51 | .02 | .67 | .02 | .57 | .02 | .65 | .02 | .55 | .02 | .60 | .02 | .54 | .02 | .63 | .02 | .56 | .03 |
|  | FA |  |  |  |  |  |  |  |  |  |  |  |  |  |  |  |  |  |  |  |  |  |
|  |  | LF | .10 | .01 | .16 | .01 | .10 | .01 | .15 | .01 | .11 | .01 | .21 | .01 | .14 | .01 | .25 | .02 | .19 | .02 | .29 | .02 |
|  |  | HF | .20 | .02 | .27 | .02 | .24 | .02 | .28 | .02 | .24 | .02 | .35 | .02 | .25 | .02 | .38 | .03 | .31 | .03 | .39 | .03 |

*Note*. M = mean; SE = standard errors; FA = false alarms; LF = low-frequency words; and HF = high-frequency words.

** *Supplementary Figure.* All 3 experiments: Recognition memory performance across the lifespan. A) Pr scores. B) Estimates of recollection. C) Estimates of familiarity. Error bars represent standard errors. LF = low-frequency words; HF = high-frequency words.
